# Mycobacterial Hsp65 antigen delivered by invasive *Lactococcus lactis* reduces intestinal inflammation and fibrosis in TNBS-induced chronic colitis model

**DOI:** 10.1038/s41598-020-77276-8

**Published:** 2020-11-18

**Authors:** Vanessa Pecini da Cunha, Tatiane Melo Preisser, Mariana Passos Santana, Denise Carmona Cara Machado, Vanessa Bastos Pereira, Anderson Miyoshi

**Affiliations:** 1 Laboratory of Genetic Technology, Department of Ecology, Genetics and Evolution, Institute of Biological Sciences, Federal University of Minas Gerais, Belo Horizonte, Minas Gerais Brazil; 2 Center for Gastrointestinal Biology, Department of Morphology, Institute of Biological Sciences, Federal University of Minas Gerais, Belo Horizonte, Minas Gerais Brazil

**Keywords:** Biotechnology, Microbiology, Molecular biology

## Abstract

Intestinal fibrosis associated with Crohn’s disease (CD), which a common and serious complication of inflammatory bowel diseases. In this context, heat shock proteins (HSPs) might serve as an alternative treatment because these antigens play important roles in the regulation of effector T cells. We thus evaluated the anti-inflammatory and antifibrotic capacities of an invasive and Hsp65-producing strain—*Lactococcus lactis* NCDO2118 FnBPA+ (pXYCYT:Hsp65)—in chronic intestinal inflammation to assess its potential as an alternative therapeutic strategy against fibrotic CD. Experimental colitis was induced by 2,4,6-trinitrobenzene sulfonic acid (TNBS) in BALB/c mice, and the mice were treated orally with *L. lactis* NCDO2118 FnBPA+ (pXYCYT:Hsp65) via intragastric gavage. The oral administration of this strain significantly attenuated the severity of inflammation and intestinal fibrosis in mice (*p* < 0.05). These results are mainly justified by reductions in the levels of the pro-fibrotic cytokines IL-13 and TGF-β and increases in the concentration of the regulatory cytokine IL-10. The *L. lactis* NCDO2118 FnBPA+ (pXYCYT:Hsp65) strain contributed to reductions in the severity of inflammatory damage in chronic experimental CD, and these findings confirm the effectiveness of this new antifibrotic strategy based on the delivery of therapeutic proteins to inside cells of the host intestinal mucosa.

## Introduction

Inflammatory bowel diseases (IBDs) are a set of disorders that affect the gastrointestinal tract, and their incidence and prevalence rates have been increasing globally in the twenty-first century^1^. IBDs essentially comprise two diseases, Crohn’s disease (CD) and ulcerative colitis (UC), which are characterized by chronic and recurrent inflammation of the mucosa^2^.

CD is an incurable syndrome whose main symptom is abdominal pain associated with diarrhea, fever, fatigue, weight loss and weakness due to difficulty in absorbing nutrients^3,4^. In addition, one of the main complications of CD is the development of fibrosis, which results from excessive accumulation of extracellular matrix rich in collagen in the intestine layers, particularly the submucosa and muscle^5,6^.

Intestinal fibrosis related to CD occurs in approximately 30–50% of patients and can cause serious damage, such as stenosis, fistula or abscesses^7,8^. Additionally, the fibrotic complications in CD are responsible for approximately 75% of surgical resections in the first decade after the clinical diagnosis^9,10^.

Although therapies that modulate CD inflammation are currently available, there are no effective treatments to prevent or reverse fibrosis that affects carriers of the disease. In this sense, heat shock proteins (HSPs), which are important antigens in regulating effector T cells during inflammation^11,12^, can be promising candidates for the development of new antifibrogenic therapies.

Consistent with this notion, HSP60 modulates immune responses, and this modulation leads to the downregulation of Th1 responses and the upregulation of Th2 and Treg responses^13,14^, which are necessary effects for the control of CD. Additionally, the interaction between HSP60 and T cells leads to increased production and secretion of the anti-inflammatory cytokine IL-10 and reduced levels of proinflammatory cytokines, such as IFN-γ and TNF-α^15^.

In this context, our research group constructed a strain of *Lactococcus lactis* NCDO2118 that expresses *Mycobacterium leprae* Hsp65 protein^16^ through the xylose-inducible expression system (XIES)^17^. We have also demonstrated that this recombinant strain is able to completely prevent colitis in a murine model^18^.

Furthermore, oral treatment with Hsp65-secreting *L. lactis* NCDO2118 ameliorates intestinal inflammation induced by the chemical agent dextran sulfate sodium (DSS) through the induction of CD4+Foxp3+ and CD4+LAP+Treg cells and via IL-10- and Toll-like receptor 2 (TLR2)-dependent pathways^18^. The wild-type strain (*L. lactis* subsp. *lactis* NCDO2118) also exhibits anti-inflammatory and immunomodulatory properties in an inflamed colon when administered during the remission period of DSS-induced colitis^19^.

More recently, our group developed and evaluated a new recombinant strain of *L. lactis* NCDO2118 that expresses both *Staphylococcus aureus* fibronectin-binding protein A (FnBPA+) and *M. leprae* Hsp65—*L. lactis* NCDO2118 FnBPA+(pXYCYT:Hsp65)—in acute colitis^20^. This experimental strategy efficiently delivers Hsp65 to the site affected by the disease and is capable of reducing the severity of inflammatory damage caused by TNBS-induced colitis in mice. These effects are related to reduced levels of IL-12 and IL-17 and increased levels of IL-10 and secretory immunoglobulin A (sIgA)^20^.

Therefore, the present study aimed to evaluate the anti-inflammatory and antifibrotic capacities of this invasive and Hsp65-producing strain [*L. lactis* NCDO2118 FnBPA+(pXYCYT:Hsp65)] in the prevention of experimental chronic colitis chemically induced by TNBS in a murine model.

## Material and methods

### Bacterial strains, growth conditions and induction of Hsp65 expression

The invasive *L. lactis* FnBPA+ (pXYCYT:Hsp65) strain^20^ was grown at 30 °C without agitation in M17 medium (Sigma-Aldrich, São Paulo, Brazil) supplemented with 0.5% glucose, chloramphenicol (10 µg/mL) (Sigma-Aldrich, São Paulo, Brazil) and erythromycin (5 µg/mL) (Sigma-Aldrich, São Paulo, Brazil). To induce expression of the *M. leprae* Hsp65 ORF, the recombinant *L. lactis* NCDO2118 FnBPA+ (pXYCYT:Hsp65) strain was grown with 2% xylose (Dinâmica, Indaiatuba, Brazil) and 0.5% galactose (Vetec, Duque de Caxias, Brazil) for approximately 8 h (until the cells reached an OD_600 nm_ of ~ 2)^17,20^.

### Mice

BALB/c female mice (aged 6–7 weeks) were obtained from the Central Bioterium of the Federal University of Minas Gerais (UFMG, Belo Horizonte, Brazil). The mice were maintained in mini-isolators housed in ventilated racks with controlled conditions (temperature of 22 ± 2 °C, 50 ± 10% humidity, air flow of 35 exchanges/hour and 12-h light/12-h dark cycle)^20^ and were given free access to water and rodent food.

### Induction of chronic TNBS colitis

**Figure 1:**
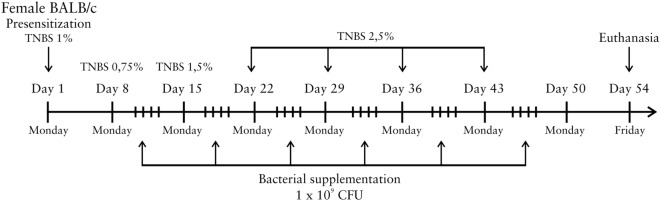
Representation of the experimental design for 2,4,6-trinitrobenzene sulfonic acid (TNBS)-induced chronic colitis. Intestinal inflammation was induced by TNBS in BALB/c µwas induced by weekly (on days 8, 15, 22, 29, 36 and 43) intrarectal administration of solutions with increasing concentrations of TNBS in 50% ethanol [0.75%, 1.5% and 2.5% TNBS (wt/vol)]. The mice received four intragastric administrations of bacterial suspension containing 1 × 10^9^ CFUs per week for 6 consecutive weeks. On day 54, all the mice were euthanized.

Chronic intestinal inflammation was induced by 2,4,6-trinitrobenzene sulfonic acid [TNBS, 5% (wt/vol) in H_2_O (Sigma-Aldrich, cat. no. p2297, São Paulo, Brazil)] using a previously described technique^21^. After a 6-h fasting period, the animals were anesthetized through an intraperitoneal injection of 100 mg/kg ketamine (Agener, São Paulo, Brazil) and 10 mg/kg xylazine (Ceva, Paulínia, Brazil). On day 1, the mice were presensitized by adding 150 µL of 1% TNBS solution dissolved in acetone/olive oil (4:1 v/v) to their shaved dorsal skin. After 7 days, chronic inflammation was induced by the weekly (on days 8, 15, 22, 29, 36 and 43) intrarectal (i.r.) administration of 100 µL of increasing concentrations of TNBS in 50% ethanol [0.75%, 1.5% and 2.5% TNBS (wt/vol)]. The mouse body weight was recorded weekly. On day 54, all the mice were sacrificed under anesthesia. A schematic of the experimental protocol used for the induction of chronic colitis is shown in Fig. 1.

### Treatment of chronic intestinal inflammation

For the treatment of chronic TNBS-induced colitis, 100 μL of the *L. lactis* FnBPA+ (pXYCYT:Hsp65) strain was administered by intragastric gavage (i.g.). Starting on the day after the intrarectal administration of TNBS solution, the mice received oral administrations of a bacterial suspension of invasive *L. lactis* FnBPA+ (pXYCYT:Hsp65) [containing 1 × 10^9^ colony forming units (CFUs) in 0.9% saline] once daily for 4 consecutive days, and these administrations were performed for 6 consecutive weeks (Fig. 1).

The experimental design included the following four groups (6 mice/group), which were established based on previous results from our research group^20^: (1) negative control of chronic colitis (C− group), the mice received 0.9% saline (i.g.) and 0.9% saline (i.r.); (2) positive control of chronic colitis (C+ group), the mice received 0.9% saline (i.g) and TNBS (i.r.); (3) NFX group, the mice were treated with invasive *L. lactis* FnBPA+ (pXYCYT:Hsp65) strain not expressing Hsp65 (i.g.) and TNBS (i.r.); and (4) NFXi group, the mice were treated with invasive *L. lactis* FnBPA+ (pXYCYT:Hsp65) strain expressing Hsp65 (i.g.) and TNBS (i.r.).

### Histopathological evaluation of intestinal inflammation and fibrosis

Each animal was given a histological score to describe the severity of chronic colitis as previously described^22^. Hematoxylin and eosin (H&E)-stained and Gomori-stained colon samples were used to obtain the histological scores of inflammation and fibrosis, respectively.

H&E-stained samples from each layer (mucosa, submucosa, muscle and serosa) of the colon wall were used to score (range of 0–4, where 4 indicates the highest severity) the severity of acute and chronic inflammation. The acute and chronic scores from each layer were summed to derive an overall inflammatory score for each mouse, and the maximum possible score was 32. The acute inflammatory score was based on hemorrhage, edema, polymorphonuclear leukocyte infiltration and necrosis, whereas the chronic inflammatory score was based on the detected number of mononuclear cells (lymphocytes and macrophages)^22^.

The Gomori-stained samples were assigned a score ranging from 0 to 5 based on the increase in collagen deposition: a score of 0 represents no increase, and scores of 1–5 represent progressive increases in collagen deposition in different layers of the colon wall. A score of 5 represents the most severe fibrosis and indicates increases in collagen throughout all layers, from the mucosa to the serosa. The fibrosis score was multiplied by 1–4 to reflect the extent (0–100%) of the section that exhibits fibrosis^22^.

### Assessment of myeloperoxidase (MPO) activity

To assess the degree of chronic inflammation, the activity of the enzyme myeloperoxidase (MPO) in colon samples was measured. Colon tissues (50 mg) were collected and homogenized in (1) cytokine extraction buffer [23.4 g of NaCl, 500 µL of Tween-20, 5 g of BSA, 34 mg of PMSF, 1 mL of DMSO, 44.6 mg of benzethonium chloride, 372 mg of Na_2_EDTA, and 40 µL of 10 mg/mL aprotinin (Sigma-Aldrich, São Paulo, Brazil) in 1 L of q.s.p. PBS1X]; (2) buffer I (pH 4.7; 0.1 M NaCl, 0.02 M Na_3_PO_4_, and 0.015 M Na_2_EDTA); and (3) 0.2% NaCl and 1.6% NaCl-5% glucose. The homogenate was then divided into two parts, and one of the parts was diluted in buffer II (pH 5.4; 0.05 M Na_3_PO_4_ and 0.5% HETAB). The homogenate was then subjected to three cycles of freezing in liquid nitrogen and thawing in water at room temperature. The substrate 3,3′,5,5′-tetramethylbenzidine (TMB) (Sigma Aldrich, São Paulo, Brazil) was then added, and the mixture was incubated at 37 °C for 5 min. Subsequently, and 0.002% H_2_O_2_ was added, and the mixture was incubated for more than 5 min at 37 °C. The reaction was interrupted by the addition of 1 M H_2_SO_4._ The color intensity was evaluated using a microplate reader (POLARIS MA616—Marconi, Piracicaba, Brazil) at 450 nm.

### Determination of the colon cytokine profile

The concentrations of pro- and anti-inflammatory cytokines in colon homogenates (100 mg of tissue/1 mL of cytokine extraction buffer) were quantified using sandwich ELISA kits [BD (BD Biosciences, San Jose, CA, USA) and R&D (R&D Systems, Minneapolis, MN, USA)] in accordance with the manufacturers’ instructions. The measured cytokines were IFN-γ, IL-12, IL-6, IL-13, IL-17, TGF-β and IL-10, and the absorbances were measured using a spectrophotometer (POLARIS MA616—Marconi, Piracicaba, Brazil) at 492 nm.

### Secretory IgA (sIgA) assay

The sIgA levels in intestinal lavage were determined using a capture ELISA. The colon was washed with 5 mL of PBS1X, and the fluid was centrifuged at 1200 rpm and 4 °C for 10 min. The sIgA levels in the supernatant were measured using goat anti-mouse IgG, human ads-UNLB and goat anti-mouse IgA conjugated with horseradish peroxidase (HRP) antibodies (Southern Biotech, Birmingham, AL, USA) according to the manufacturer's instructions. The absorbance (492 nm) was measured using a POLARIS MA616—Marconi instrument (Piracicaba, Brazil).

### Statistical analysis

The results obtained from the assessment of TNBS-induced chronic colitis are presented as the means ± standard deviations (SDs) from three experimental replicates (n = 18). All the data were analyzed by one-way analysis of variance (one-way ANOVA) followed by Tukey’s posttest using GraphPad Prism version 6.0 (GraphPad Software, San Diego, CA, USA). The values were considered statistically significant if *p* < 0.05.

### Ethical considerations

All the experiments and handling of the mice were performed in accordance with the ethical principles for animal experimentation adopted by the Animal Use Ethics Commission (CEUA, Registration Number 341/2017) of the Federal University of Minas Gerais (UFMG, Belo Horizonte, Brazil).

## Results

### Effects of *L. lactis* NCDO2118 FnBPA+ (pXYCYT:Hsp65) in clinical health parameters of mice with TNBS-induced chronic colitis

**Figure 2:**
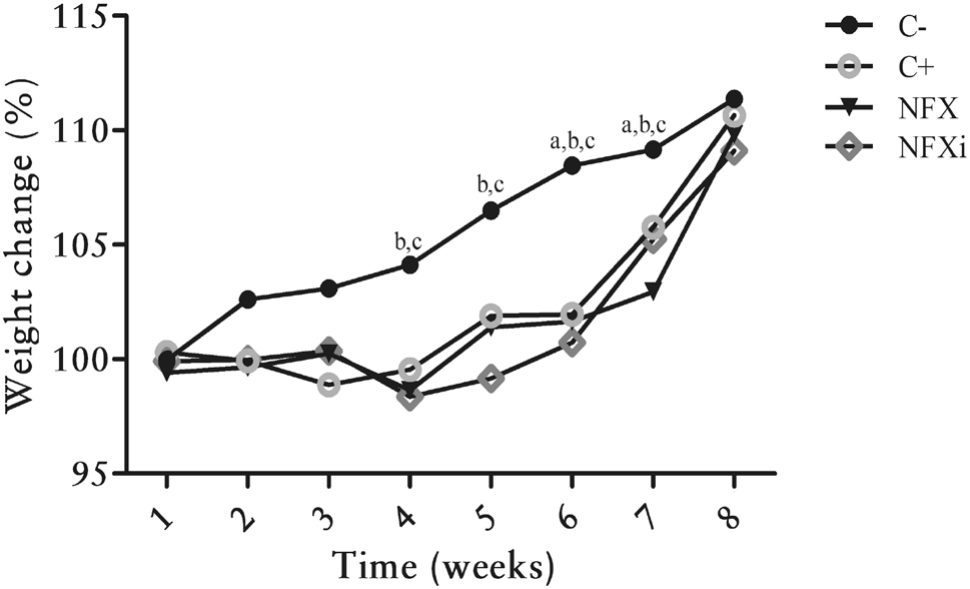
Percentage of the initial body weight of BALB/c mice with or without TNBS administration as a function of time. **a** Experimental group whose body weight percentages were significantly different from those of the C+ group. **b** Experimental group whose body weight percentages were significantly different from those of the NFX group. **c** Experimental group whose body weight percentages were significantly different from those of the NFXi group. The data are shown as the means ± SDs from three independent experiments (n = 18). *p* value: *p < 0.05.

In this study, to examine the severity of lesions caused by intrarectal TNBS administration, the body weight and histological scores were evaluated. As shown in Fig. 2, the animals subjected to TNBS administration (the NFX and NFXi groups) had lower body weights (*p* < 0.05) than those in the C− group at weeks 4 and 5. However, at weeks 6 and 7, all the mice that received TNBS (the C+, NFX and NFXi groups) presented significant weight loss (*p* < 0.05) compared with those in the C− group. No significant differences were observed among all the groups at weeks 1, 2, 3 and 8.

**Figure 3:**
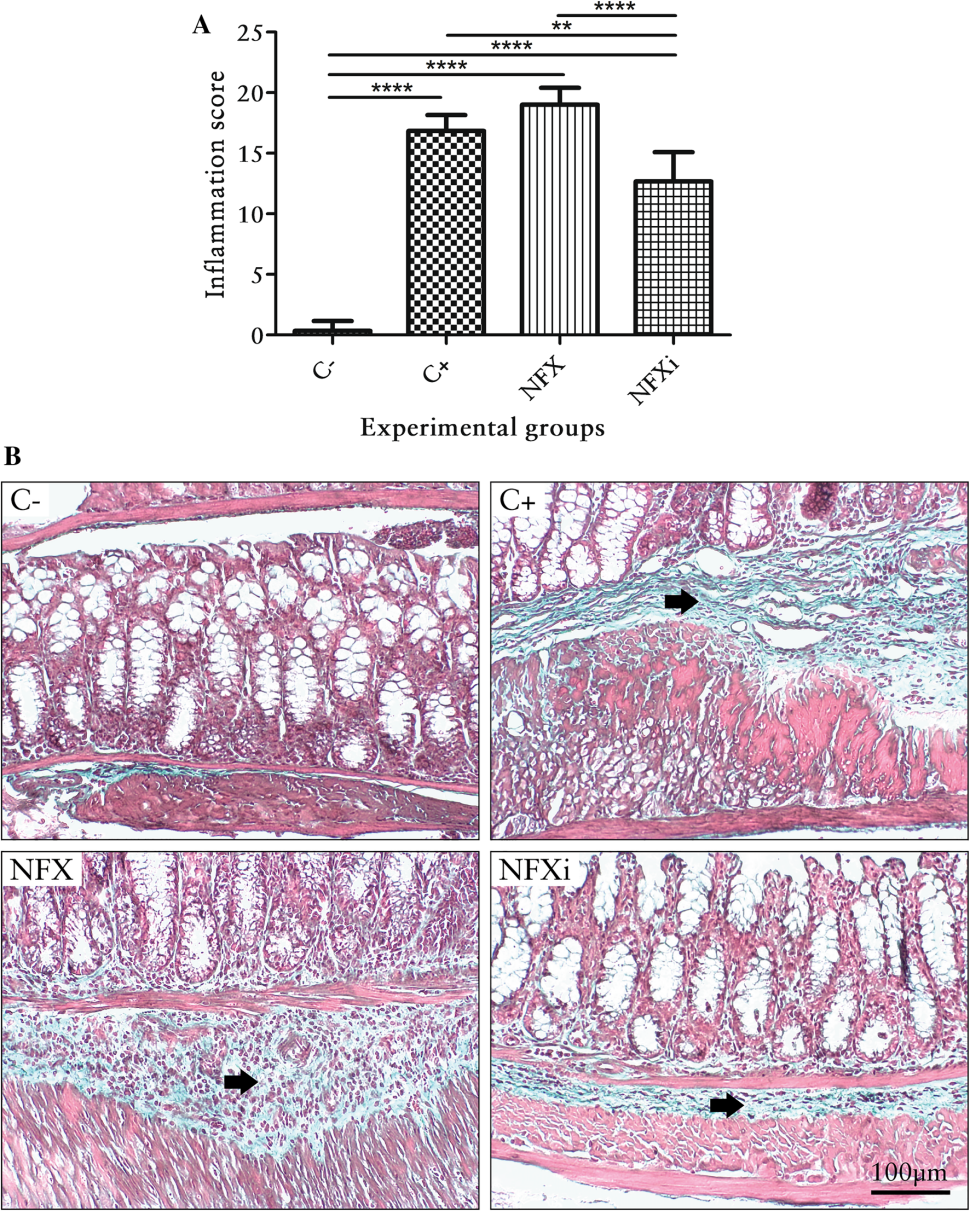
Inflammation scores and histological features of the colon of mice with chronic TNBS-induced colitis. (**A**) Inflammation scores of BALB/c mice with or without TNBS administration. (**B**) Histological features of the colon of BALB/c mice with or without TNBS administration (H&E staining). The short arrows point to the inflammatory infiltrate in the intestinal mucosa layer, and the long arrows indicate the inflammatory infiltrate in the intestinal submucosal layer. The data are shown as the means ± SDs from three independent experiments (n = 6). *p* values: **p < 0.01; ****p < 0.0001.

**Figure 4:**
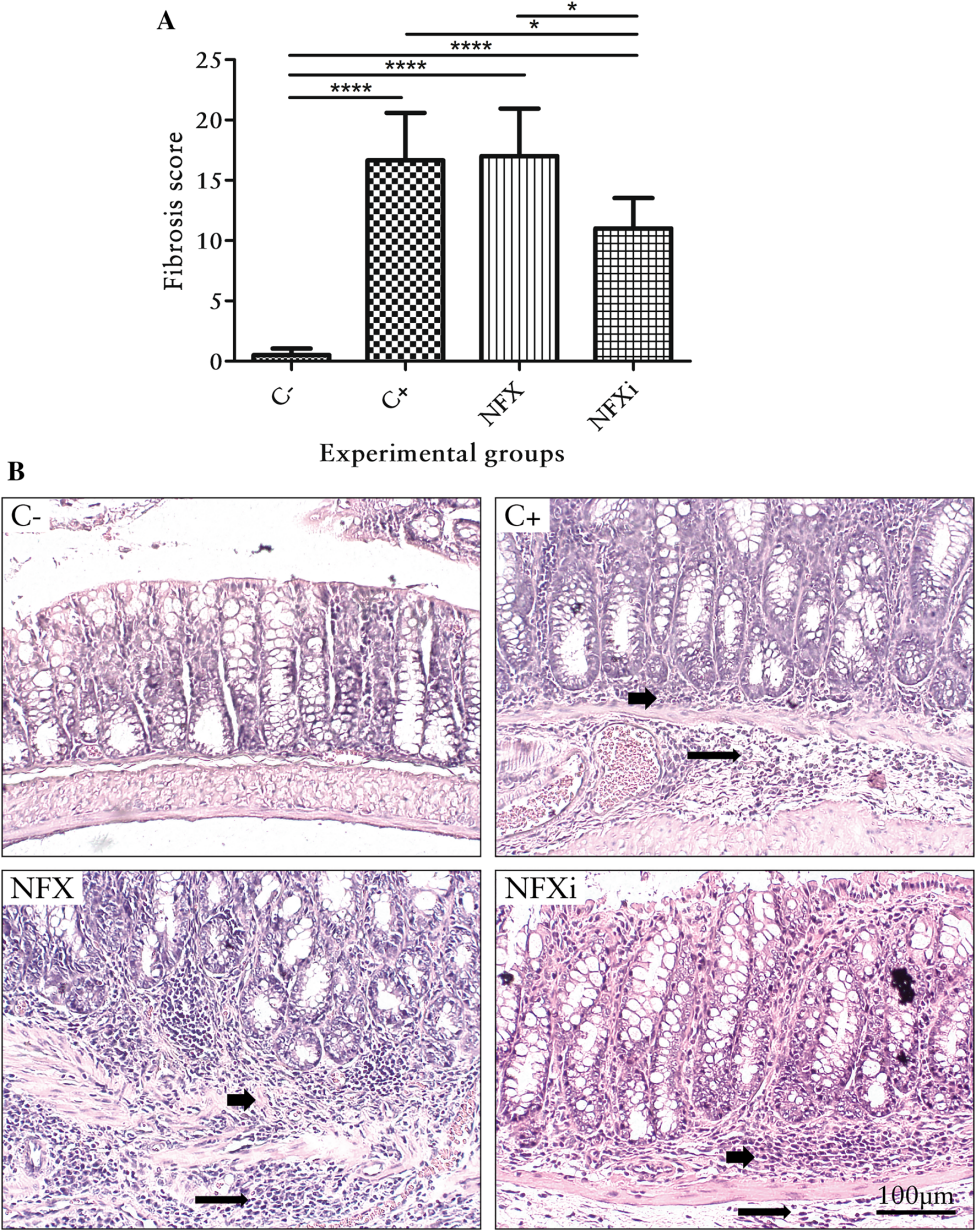
Fibrosis scores and histological features of the colon of mice with chronic TNBS-induced colitis. (**A**) Fibrosis scores of BALB/c mice with or without TNBS administration. (**B**) Histological features of the colon of BALB/c mice with or without TNBS administration (Gomori staining). The short arrows indicate fibrosis, as highlighted by the presence of collagen in the intestinal submucosal layer. The data are shown as the means ± SDs from three independent experiments (n = 6). *p* values: *p < 0.05; ****p < 0.0001.

The histological observations revealed that the mice belonging to the C− group presented neither intestinal inflammation nor fibrosis (*p* < 0.05; Figs. 3A, 4A), and H&E and Gomori staining showed that that the morphology was normal (Figs. 3B, 4B). The mucosa was intact, as demonstrated by the presence of goblet cells and the absence of erosion and inflammatory infiltrate. The submucosa was intact, as revealed by a basal collagen level and the absence of hyperemia, hemorrhage or inflammatory infiltrate. In addition, no evidence of mucosal muscle injury was observed. The muscular layer itself was intact, with no inflammatory infiltrate, collagen deposition or areas of necrosis. The serosa was thin and did not exhibit any pathological elements (Figs. 3B, 4B).

All the animals with TNBS-induced chronic colitis (with the exception of those belonging to the NFXi group) presented similar symptoms of intestinal inflammation and fibrosis (Figs. 3A, 4A). H&E and Gomori staining showed pathological changes in all the layers (Figs. 3B, 4B). The mucous layer contained areas of erosion and inflammatory infiltrate. The submucosal layer presented hyperemic areas with mild hemorrhage and inflammatory infiltrate with polymorphonuclear and mononuclear leukocytes at degrees ranging from severe to very severe. The muscular layer itself showed areas of necrosis and inflammatory infiltrate, and the serous layer was marked by inflammatory infiltrate (Fig. 3B). Fibrosis was present in all the layers and was predominant in the submucosal layer (Fig. 4B).

The NFXi group exhibited significantly reduced intestinal inflammation (*p* < 0.05) compared with the animals belonging to the C− and NFX groups (Fig. 3A). Additionally, this group presented significantly reduced fibrosis (*p* < 0.05) compared with the other groups (C+ and NFX groups) that were subjected to TNBS administration (Fig. 4A). Thus, H&E and Gomori staining showed that the NFXi group presented lesions characterized as mild based on the intensity of acute inflammatory infiltrate, hyperemia, erosion, necrosis and fibrosis (Figs. 3B, 4B).

### Effects of *L. lactis* NCDO2118 FnBPA+ (pXYCYT:Hsp65) on inflammatory markers in mice with TNBS-induced colitis

To investigate the effect of MPO activity, which serves as a biomarker for the influx of neutrophils to the inflammatory site, we assessed the ability of *L. lactis* NCDO2118 FnBPA+ (pXYCYT:Hsp65) to decrease inflammatory cell infiltration in colon samples. No difference in MPO activity was observed among the studied groups.

### Effects of *L. lactis* NCDO2118 FnBPA+ (pXYCYT:Hsp65) on the secretion of pro‐and anti‐inflammatory cytokines in mice with TNBS-induced colitis

To assess whether *L. lactis* NCDO2118 FnBPA+ (pXYCYT:Hsp65) can modulate the cytokine levels in chronic colitis, the levels of IFN-γ, IL-12, IL-6, IL-13, IL-17, TGF-β and IL-10 in colonic tissues were measured. The levels of IFN-γ and IL-12, which are cytokines related to the Th1 immune profile, did not differ among the experimental groups.

**Figure 5:**
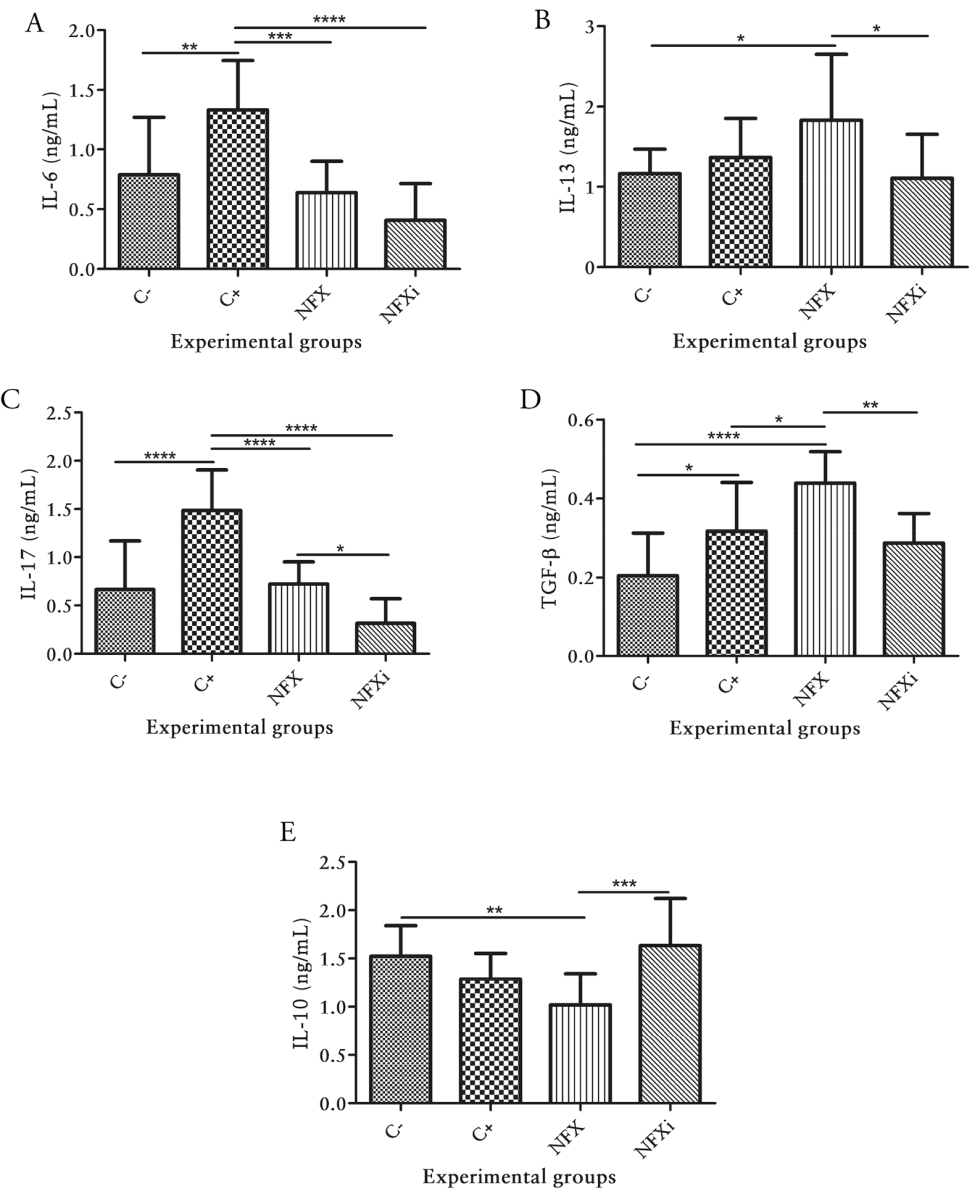
Cytokine levels in BALB/c mice with or without TNBS administration. The concentration of (**A**) Interleukin-6 (IL-6), (**B**) Interleukin-13 (IL-13), (**C**) Interleukin-17 (IL-17), (**D**) Transforming growth factor-(TGF-β) and (**E**) Interleukin-10 (IL-10), were determined in colon homogenates using ELISA. The data are shown as the means ± SDs from three independent experiments (n = 12). *p* values: *p < 0.05; **p < 0.01; ***p < 0.001; ****p < 0.0001.

In contrast, the evaluation of Th2 profile-related cytokines revealed that the NFXi group presented significantly lower IL-6 levels (*p* < 0.05) than those in the C+ group (Fig. 5A) and significantly lower IL-13 levels (*p* < 0.05) than those in the NFX group (Fig. 5B).

The assessment of the IL-17 levels showed that the animals in the NFXi group had significantly lower IL-17 levels (*p* < 0.05) than the animals in the C+ and NFX groups (Fig. 5C). The measurement of anti-inflammatory cytokines revealed that the NFXi group presented significantly decreased TGF-β levels (*p* < 0.05; Fig. 5D) and significantly increased IL-10 levels (*p* < 0.05; Fig. 5E) than those in the NFX group.

### Effects of *L. lactis* NCDO2118 FnBPA+ (pXYCYT:Hsp65) on secretory IgA (sIgA) in chronic colitis

The levels of sIgA in intestinal lavage were then determined to investigate whether the oral administration of *L. lactis* NCDO2118 FnBPA+ (pXYCYT:Hsp65) could alter the production of sIgA in the colon. No differences in the sIgA concentrations were observed among the groups.

## Discussion

CD, a multifactorial IBD for which there is no cure, is characterized by chronic inflammation of the digestive system wall^10,23^, and intestinal fibrosis is a recurrent complication of CD that can lead to serious damage, such as intestinal obstruction^8^. Therefore, in the present study, we used a mouse model of chronic colitis to evaluate the anti-inflammatory and antifibrotic potential of an invasive and Hsp65-producing strain [*L. lactis* NCDO2118 FnBPA+ (pXYCYT:Hsp65)]. Mycobacterial Hsp65 protein produced by *L. lactis* NCDO2118 has been described as a molecule with therapeutic potential in C57BL/6 mice with DSS-induced intestinal inflammation^18^ and in BALB/c mice with TNBS-induced colitis^20^.

Initially, we adopted a model of chronic colitis chemically induced by TNBS because the onset of inflammation is immediate, the procedure is relatively simple, and the induction produces a chronic inflammatory lesion associated with intestinal tissue fibrosis^21,24^. Additionally, this model allows us to monitor important animal health parameters, such as the body weight and histological features of acute and chronic inflammation and fibrosis.

The analysis of the body weight showed that all the animals that received TNBS (C+, NFX and NFXi groups) exhibited weight loss compared with those in the C− group. This finding was obtained because this model of chronic colitis promotes transmural inflammation in the animals, which is followed by severe diarrhea, weight loss and rectal prolapse^25^. In addition, at week 8 in the experimental schedule, when the administrations of intrarectal TNBS were completed, all the animals belonging to these three groups exhibited some recovery, and their body weights were nearly equal to that of the C− group. These results might be explained by the decreased ability to maintain TNBS-induced colitis in mice older than 8 weeks^26^.

The analysis of the histological scores of inflammation and fibrosis revealed that the animals in the NFXi group exhibited a significant decrease in the severity of colitis compared with those in the C+ and NFX groups, which emphasized the therapeutic potential of the *L. lactis* NCDO2118 FnBPA+ (pXYCYT:Hsp65) strain. In a similar study, we showed that this new recombinant strain can attenuate the severity of intestinal inflammation in acute TNBS-induced colitis by increasing the levels of the anti-inflammatory cytokine IL-10 and immunoglobulin sIgA^20^.

We measured MPO as an inflammatory biomarker of colitis because an increased concentration of MPO indicates the infiltration of activated neutrophils into inflamed tissue and suggests an exacerbation of inflammation in the colon^27^. In the present study, significant differences could not be detected among the groups, even though the release of MPO is common in both acute and chronic inflammation^28^.

In addition, no significant differences in the proinflammatory cytokines IFN-γ and IL-12 were found among the groups, and this finding was most likely due to the fact that these cytokines were assessed on day 54 of the experimental schedule, which is after the peak in cytokine production. Consistent with our observation, the literature reports that these cytokines (IFN-γ and IL-12) participate in the acute process of intestinal inflammation induced by TNBS that their peak production thus occurs around day 7 in BALB/c mice^24^.

In the inflamed mucosa of patients with IBDs, the interleukins IL-6 and IL-13, which are cytokines related to the Th2 profile, are commonly found at increased levels^2,29^. The assessment of the IL-6 levels performed in this study showed that the NFXi group presented reduced levels of this proinflammatory cytokine compared with the C+ group. IL-6 has been identified as an important interleukin that mediates chronic inflammation in colitis^30,31^, whereas IL-13 is a potent inducer of fibrosis in chronic autoimmune diseases, including CD^8,32^. In this study, significant differences in the IL-13 levels were found between the NFXi and NFX groups. These results corroborate the histological findings of fibrosis, which revealed that the mice in the NFXi group presented less collagen deposition in the intestinal submucosa.

Because TNBS-induced colitis is characterized by a Th1/Th17 immune profile^33^, the levels of the cytokine IL-17 were measured. The IL-17 results showed a reduction in this proinflammatory cytokine in the NFXi group compared with those the C+ and NFX groups. This reduction is important for achieving decreases in histological changes related to both acute and chronic inflammation and fibrosis because IL-17 exerts profibrotic functions in the intestinal mucosa^8,34^.

The anti-inflammatory cytokines TGF-β and IL-10 were also measured. Although TGF-β is considered an anti-inflammatory cytokine^35^, no significant increase in the level of this cytokine was found in the present study in the NFXi group. Nonetheless, high concentrations of TGF-β, together with IL-13, can contribute to the increase in tissue fibrosis observed in IBDs^32^, and previous studies have shown the profibrogenic role of TGF-β in chronic colitis^9,30,36,37^. Here, the decreases in TGF-β observed in the NFXi group compared with the levels in the C+ and NFX groups, which are associated with the above-mentioned decreases in IL-13, might have contributed to the observed reductions in the intestinal inflammation and fibrosis scores.

Our analysis of IL-10, which is the main regulatory cytokine for inflammation, showed a significant increase in the NFXi group compared with the NFX group. This result possibly explains the inflammation scores and decreased histological features of the NFXi group. Notably, IL-10 is an influential interleukin that can modulate pro- and anti-inflammatory cytokines^38^, and IL-10 can also suppress exacerbated mucosal immune responses, maintain intestinal homeostasis and tolerance to commensal microbiota^39^ and inhibit collagen deposition in the host intestine^30^.

The intestinal sIgA levels were also measured, but no significant differences were found among the groups. In mammals, particularly mice and humans, IgA is the most abundant antibody. sIgA can protect the intestinal mucosa against toxins and infection^40^. Thus, *L. lactis* NCDO2118 FnBPA+ (pXYCYT:Hsp65) did not affect the increased sIgA levels in the chronic colitis model.

In conclusion, the results demonstrate that the oral administration of *L. lactis* FnBPA + (pXYCYT:Hsp65) effectively improves various health parameters of animals with chronic colitis. Notably, the effectiveness of this new experimental strategy can be explained by the observed reductions in the severity of inflammation and intestinal fibrosis through decreases in IL-13 and TGF-β and increases in IL-10. Therefore, this strategy of delivering therapeutic proteins to mammalian cells should be considered an alternative approach for maintaining the pro- and anti-inflammatory balance of the gastrointestinal tract in individuals affected by fibrotic CD.
